# Facile Synthesis of a Croconaine‐Based Nanoformulation for Optoacoustic Imaging and Photothermal Therapy

**DOI:** 10.1002/adhm.202002115

**Published:** 2021-03-18

**Authors:** Nian Liu, Patrick O'Connor, Vipul Gujrati, Dimitris Gorpas, Sarah Glasl, Andreas Blutke, Axel Walch, Karin Kleigrewe, Michael Sattler, Oliver Plettenburg, Vasilis Ntziachristos

**Affiliations:** ^1^ Chair of Biological Imaging Center for Translational Cancer Research (TranslaTUM) Technical University of Munich Munich 81675 Germany; ^2^ Institute of Biological and Medical Imaging Helmholtz Zentrum München (GmbH) Neuherberg 85764 Germany; ^3^ Institute of Medicinal Chemistry Helmholtz Zentrum München (GmbH) Neuherberg 85764 Germany; ^4^ Institute of Structural Biology Helmholtz Zentrum München (GmbH) Neuherberg 85764 Germany; ^5^ Research Unit Analytical Pathology Helmholtz Zentrum München (GmbH) Neuherberg 85764 Germany; ^6^ Bavarian Center for Biomolecular Mass Spectrometry (BayBioMS) Technical University of Munich Freising 85354 Germany; ^7^ Bavarian NMR Center and Center for Integrated Protein Science Munich Technical University of Munich Garching 85747 Germany; ^8^ Center for Biomolecular Research Institute of Organic Chemistry Leibniz Universität Hannover Hannover 30167 Germany

**Keywords:** croconaine, nanoparticles, optoacoustics, photothermal therapy

## Abstract

Near‐infrared (NIR) light absorbing theranostic agents can integrate optoacoustic imaging and photothermal therapy for effective personalized precision medicine. However, most of these agents face the challenges of unstable optical properties, material‐associated toxicity, and nonbiodegradability, all of which limit their biomedical application. Several croconaine‐based organic agents able to overcome some of these limitations have been recently reported, but these suffer from complicated multistep synthesis protocols. Herein, the use of CR760, a croconaine dye with excellent optical properties, is reported for nanoparticle formulation and subsequent optoacoustic imaging and photothermal therapy. Importantly, CR760 can be conveniently prepared in a single step from commercially available materials. Furthermore, CR760 can be covalently attached, via a polyethylene glycol linker, to the *α*_v_ *β*_3_ integrin ligand c(RGDyC), resulting in self‐assembled nanoparticles (NPs) with cancer‐targeting capability. Such CR760RGD‐NPs exhibit strong NIR absorption, high photostability, high optoacoustic generation efficiency, and active tumor‐targeting, making them ideal candidates for optoacoustic imaging. Due to favorable electron transfer, CR760RGD‐NPs display a 45.37% photothermal conversion efficiency thereby rendering them additionally useful for photothermal therapy. Targeted tumor elimination, biosafety, and biocompatibility are demonstrated in a 4T1 murine breast tumor model. This work points to the use of CR760RGD‐NPs as a promising nanoagent for NIR‐based cancer phototheranostics.

## Graphical abstract

CR760, a croconaine dye with excellent optical properties, is synthesized in a single step and subsequently nanoformulated for optoacoustic imaging and photothermal therapy of cancer.

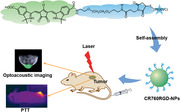

## 1 Introduction

Theranostic agents that integrate real‐time diagnosis with a therapeutic effect have the potential to accurately target diseased tissues and optimally exert therapy, representing a promising and cost‐effective type of precision medicine.^[^ ^1^ ^]^ Among the reported theranostics agents, near‐infrared (NIR) absorbing agents are characterized by a simple but effective design: these agents only utilize light to generate optical or ultrasound signals for diagnosis while simultaneously liberating either heat or reactive oxygen species (ROS), resulting in tumor elimination.^[^ ^2^ ^]^ A wide spectrum of biocompatible NIR absorbing organic dye‐based agents have been recently explored for enhancing contrast in optoacoustic imaging and for photothermal therapy (PTT).^[^ ^3^ ^]^ Optoacoustic imaging can visualize tumor tissue at centimeter depths with high contrast and spatial resolution,^[^ ^4^ ^]^ and PTT has the advantages of minimal invasiveness, spatiotemporal selectivity, and reduced side effects.^[^ ^5^ ^]^

Croconaine dyes have been suggested to overcome common limitations of other organic dyes considered for optoacoustic imaging and PTT.^[^ ^6^ ^]^ In particular, croconaine dyes improve on the thermal stability, photobleaching resistance, solvatochromism, and spectral tenability shortcomings of most common organic dyes, such as cyanine, squaraine, BODIPY, and tetrapyrroles.^[^ ^7^ ^]^ Nevertheless, most of the proposed croconaine derivatives suffer from H‐aggregation, modest chemical instability, and low solubility and are therefore not suitable for in vivo applications.^[^ ^8^ ^]^ There is therefore a need for a croconaine dye with improved properties.

Some of these challenges, such as the structural stacking and dye aggregation of croconaine dyes, can be addressed by chemical modifications. Smith et al. reported a stearic interaction between a pH‐sensitive croconaine dye (Croc) and rotaxane, which prevented the aggregation of Croc.^[^ ^9^ ^]^ The interlocked Croc‐rotaxane complex was loaded into liposomes and used for optoacoustic imaging applications.^[^ ^9^ ^]^ However, synthesis of Croc and rotaxane consists of a multistep protocol. In another approach, Song et al. prevented dye aggregation via electronic effect by incorporating anionic carboxylates into croconaine‐derived CR780,^[^ ^10^ ^]^ which was followed by its modification with polyethylene glycol (PEG) and use in optoacoustic and phototherapy applications.^[^ ^11^ ^]^ However, the synthesis and purification of CR780 also involve multistep protocols.

Here, we use the croconaine‐based dye CR760 that, similar to Croc and CR780, provides superior theranostic properties compared to most existing croconaine derivatives and, unlike any other croconaine derivative, can also be easily synthesized via a single‐step protocol. We report for the first time the development of PEGylated CR760‐based dye nanoparticles for theranostic (optoacoustic imaging and phototherapy) applications. The croconaine‐derived CR760 structure was previously reported for photovoltaic cell applications.^[^ ^12^ ^]^ Importantly, we found that CR760 has no aggregation, blueshift, or chemical instability issues and is easy to chemically modify. Moreover, CR760 can be synthesized and purified in a single step. Therefore, we conjugated CR760 with NH_2_‐PEG2000‐SH and c(RGDyC) peptides to generate safe and biocompatible self‐assembled nanoparticles (CR760RGD‐NPs) with tumor‐targeting capability by supramolecular assembly.^[^ ^13^ ^]^ CR760RGD‐NPs exhibit a strong absorption peak at 760 nm (no quenching), high optoacoustic generation efficiency (OGE), high photothermal conversion efficiency (PCE) (45.37%) and photostability as compared with the gold standard Indocyanine Green (ICG) approved by the United States Food and Drug Administration (FDA). Modification with c(RGDyC) increased the effect of CR760 on tumor‐specific binding and accumulation. In summary, we report CR760‐derived nanoparticles as promising and highly efficient phototheranostic agents for cancer diagnosis and therapy.

## 2 Results

### 2.1 Synthesis and Characterization of CR760

CR760 is synthesized in a single‐step in which the croconaine backbone is modified with a strong electron donor (D) and two strong acceptors (A) to form a “D−A−D” structure. Here, the condensation reaction of croconic acid (D) and 2,3,3‐Trimethyl‐3H‐indole‐5‐carboxylic acid (A) resulted in a yield of 81% (Figure S1, Supporting Information). The chemical structure of CR760 was characterized by ^1^H NMR spectroscopy and matrix‑assisted laser desorption/ionization time of flight (MALDI‐TOF) mass spectrometry (Figure S2, Supporting Information). CR760 has moderate solubility in common organic solvents, such as chloroform, dichloromethane, and ethanol (Figure S3, Supporting Information). Table S1 (Supporting Information) shows the experimental comparison of photophysical properties of CR760 with the commercially available IRDye800CW and the gold industry standard FDA‐approved ICG. Comparative data show that CR760 has a strong absorption peak at 760 nm and exhibits a significantly higher molar absorption coefficient and a much smaller quantum yield than ICG and IRDye800CW in ethanol. Furthermore, the OGE of CR760, ICG, and IRDye800CW were measured in 10% fetal bovine serum (FBS). The OGE value reflects the ability of a compound to convert optically absorbed energy into pressure waves, which in turn provides information regarding optoacoustic intensity. CR760 exhibits an excellent OGE which is 1.75 times higher than that of ICG and 3.2 times higher than that of IRDye800CW (Figure S4 and Table S1, Supporting Information).

### 2.2 Synthesis and Characterization of CR760RGD‐NPs

**Figure 1:**
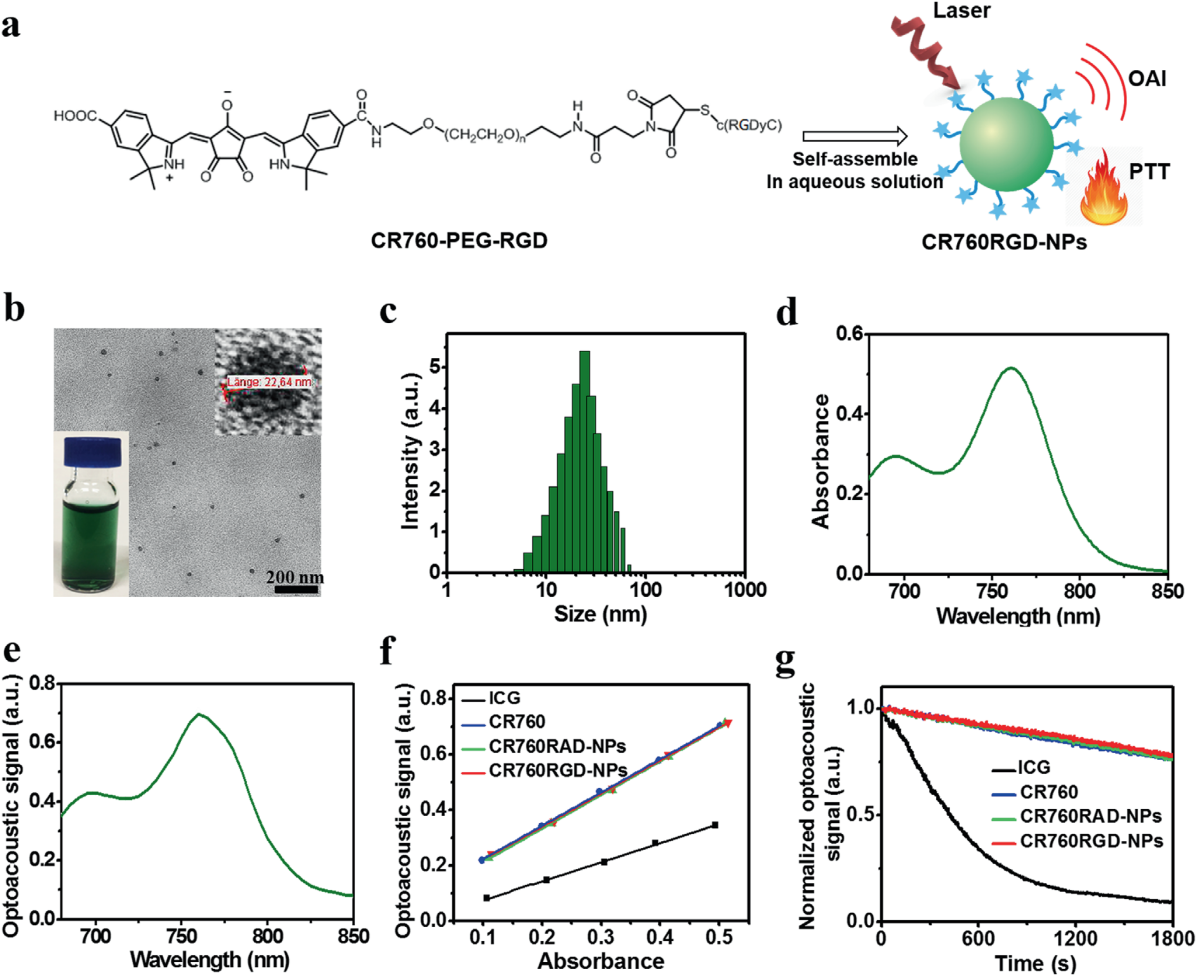
Synthesis and characterization of CR760RGD‐NPs. a) Schematic illustration of the preparation of CR760RGD‐NPs for optoacoustic imaging and photothermal therapy (PTT). b,c) TEM image and dynamic light scattering (DLS) profile of CR760RGD‐NPs (insets represent the magnified TEM image and digital photograph of CR780RGD‐NPs in water). d,e) Optical spectrum and optoacoustic spectrum of CR760RGD‐NPs. f) Optoacoustic signal intensity of ICG, CR760, CR760RAD‐NPs, and CR760RGD‐NPs at different concentrations. g) Optoacoustic signal degradation of ICG, CR760, CR760RAD‐NPs, and CR760RGD‐NPs after pulsed laser irradiation (fluence 10 mJ cm^−2^, 30 min).

Self‐assembled nanoparticles were prepared by mono‐amide conjugation of the bis‐carboxylic acid CR760 with NH_2_‐PEG2000‐SH followed by Michael addition of the free thiol to the tumor‐targeting peptide c(RGDyC) (**Figure** **1**a). Although the bis‐amide was easier to synthesize, this led to an inferior dye that was blueshifted by 53 nm relative to the monoamide. Density functional theory calculations were used to rationalize the beneficial effect of anionic substituents on the chromophore (Figure S5, Supporting Information). Excitation energies were predictably overestimated,^[^ ^14^ ^]^ however the calculations did corroborate our experimental finding of a blueshift of the neutral bis‐amide relative to the anionic monoamide, likely due to a lesser degree of charge transfer in the singlet excitation state of the bis‐amide. A negative control group consisting of CR760RAD‐NPs was also synthesized in a similar fashion utilizing a c(RADyC) peptide, which has a molecular weight comparable to the c(RGDyC) peptide but no active targeting ability toward integrin *α*_V_ *β*_3_. Figure S6 (Supporting Information) shows the CR760‐PEG‐RAD and CR760‐PEG‐RGD synthesis steps, with the corresponding MALDI‐TOF analyses indicating the purity of the compounds (Figure S7, Supporting Information). Figure 1b shows the self‐assembled CR760RGD‐NPs as a clear olive‐green, homogeneous aqueous solution. The shape and size of the nanoparticles were evaluated by transmission electron microscopy (TEM) and dynamic light scattering (DLS). Figure 1b,c shows that the CR760RGD‐NPs are self‐assembled nanosized aggregates with an average diameter of ≈23 nm. The CR760RAD‐NPs control groups showed a similar size distribution (Figure S8, Supporting Information). Figure 1d,e shows the optical and optoacoustic spectra of CR760RGD‐NPs. A narrow and intense peak at 760 nm was similar to data obtained for CR760RAD‐NPs (Figure S9, Supporting Information). The zeta potential of CR760RAD‐NPs and CR760RGD‐NPs is −15.8 and −16.3 mV, respectively (Figure S10, Supporting Information). Next, the original optoacoustic phantom images (Figure S11, Supporting Information) were corrected with India ink and normalized with Brilliant Black BN. Figure 1f demonstrates that the OGE of CR760RGD‐NPs is 1.75 times higher than that of ICG, indicating that CR760RGD‐NPs can function as an efficient optoacoustic imaging agent. Furthermore, the photostability of CR760RGD‐NPs was evaluated by irradiating the samples continuously with a pulsed laser (fluence 10 mJ cm^−2^) for 30 min and benchmarking against ICG. Figure 1g shows that both CR760‐derived nanoparticles are photostable in comparison to ICG, which under the same conditions was photobleached by up to 94%. Additionally, CR760RGD‐NPs exhibit enhanced fluorescence compared to free CR760, due to PEGylation and self‐assembly improving the dispersibility of hydrophobic CR760 (Figure S12, Supporting Information).^[^ ^11^, ^15^ ^]^ Next, ROS generation of CR760RGD‐NPs was assessed by 9,10‐dimethylnathracene (DMA).^[^ ^16^ ^]^ Figure S13 (Supporting Information) shows no decrease of DMA fluorescence intensity in CR760RGD‐NP samples after laser irradiation, indicating no ^1^O_2_ generation and no photodynamic effect. These results of characterization studies clearly indicate that the CR760RGD‐NPs self‐assemble into ultrasmall nanoparticles which exhibit a high OGE and high photostability, and as such are suitable for in vivo optoacoustic imaging and PTT.

### 2.3 In Vitro Photothermal Effect of CR760RGD‐NPs

**Figure 2:**
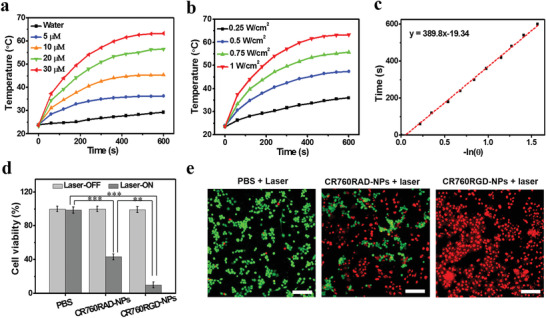
In vitro photothermal effect of CR760RGD‐NPs. a) Temperature change curves of CR760RGD‐NPs at different concentrations upon exposure to a 780 nm CW laser (1 W cm^−2^). b) Temperature change curves of CR760RGD‐NPs (30 × 10^−6^ m) upon exposure to a 780 nm CW laser at different power densities. c) Plot of time as a function of −ln(*θ*) for the raw data and a linear fit during cooling after 10 min of irradiation as described for b). d) Relative viabilities of 4T1 cells after treatment with PBS, CR760RAD‐NPs (20 × 10^−6^ m), and CR760RGD‐NPs (20 × 10^−6^ m) with (or without) 780 nm CW laser irradiation at 1 W cm^−2^ for 5 min (Two‐Way ANOVA with Tukey's HSD test, **P < 0.01, ***P < 0.001). e) Fluorescence images of live/dead staining of 4T1 cells after different treatments. Scale bars, 100 µm.

To assess the PTT potential of CR760RGD‐NPs, increasing concentrations of CR760RGD‐NPs at 0, 5, 10, 20, or 30 × 10^−6^ m were exposed to a 780 nm CW laser at a power density of 1.0 W cm^−2^. **Figure** **2**a shows a concentration‐dependent heating effect. Irradiation of a 30 × 10^−6^ m solution of CR760RGD‐NP generated a 39.9 °C change, which compared favorably to the 12.0 °C change observed for the water control. Preclinical studies of PTT therapeutics show that heating cancer cells to 42–50 °C for more than 5 min can be cytotoxic.^[^ ^3a^ ^]^ The effect of laser power intensity (0.25, 0.5, 0.75, or 1.0 W cm^−2^) on the temperature change of CR760RGD‐NPs (30 × 10^−6^ m) was explored next. Figure 2b shows a clear laser power intensity dependence on the temperature increase. The PCE of CR760RGD‐NPs was calculated using CR760RGD‐NPs irradiated with 780 nm CW laser for 10 min and cooled to room temperature. The temperature changes were recorded using the IR thermal camera and PCE was calculated to be 45.37% (Figure 2c).

To confirm if the targeting c(RGDyC) peptide is exposed on the surface of the nanoparticles, we carried out a cell uptake assay by confocal imaging in vitro. To monitor the difference in cell targeting ability, we compared differences in the uptake of targeted nanoparticles (CR760RGD‐NPs) with nontargeted nanoparticles (CR760RAD‐NPs) after a 4 h incubation in 4T1 cells. Significant differences in cell uptake efficiency were observed between the two groups, with an enhanced cell uptake of CR760RGD‐NPs observed due to the active targeting ability (Figure S14, Supporting Information). To further evaluate the PTT potential of CR760RGD‐NPs, photocytotoxicity of CR760RGD‐NPs on 4T1 cells after a 4 h incubation was evaluated using a standard MTT cell vaibility assay. CR760RGD‐NPs did not generate ROS in the process of laser irradiation in vitro (Figure S15, Supporting Information), indicating that the photocytotoxicity of CR760RGD‐NPs mainly arises from the photothermal effect.^[^ ^17^ ^]^ Figure 2d shows that both CR760RAD‐NPs and CR760RGD‐NPs show negligible cytotoxicity at a concentration of up to 20 × 10^−6^ m. However, upon 5 min of irradiation with a 780 nm CW laser at 1.0 W cm^−2^, the viability of cells treated with CR760RGD‐NPs was only 9.5%, while the viability of cells treated with CR760RAD‐NPs was 41.2%. These results point to the enhanced tumor targeting and accumulation of CR760RGD‐NPs compared to the CR760RAD‐NP control. To visualize the PTT effect of CR760RGD‐NPs, the viability of 4T1 cells was determined by live/dead cells assays. 4T1 cells incubated with phosphate‐buffered saline (PBS), CR760RAD‐NPs, and CR760RGD‐NPs before and after laser irradiation were costained with calcein‐acetoxymethyl (calcein‐AM, green color marking live cells) and ethidium homodimer‐1 (EthD‐1, red color marking dead cells). The difference in cellular cytotoxicity when incubated with the control and CR760RGD‐NPs is clearly evident, with the latter displaying the most pronounced cytotoxic effect (Figure 2e).

### 2.4 In Vivo Optoacoustic Imaging with CR760RGD‐NPs

**Figure 3:**
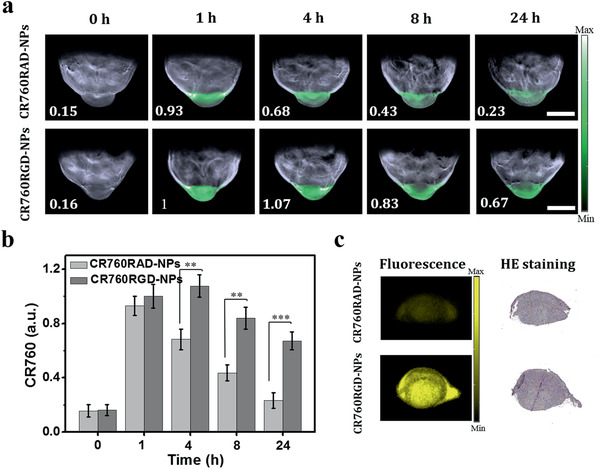
In vivo multispectral optoacoustic tomography (MSOT) imaging of CR760RAD‐NPs and CR760RGD‐NPs. a) Representative unmixed MSOT images of 4T1 subcutaneous tumor models i.v. injected with 100 µL of CR760RAD‐NPs or CR760RGD‐NPs (1 × 10^−3^ m), scale bar = 5 mm (*n* = 5). Unmixed CR760 signal is shown by the green color in the tumor region. b) Quantification of panel a): CR760RAD‐NPs and CR760RGD‐NPs concentrations in the tumor region measured over time (*n* = 5, One‐Way ANOVA with Tukey's HSD test, **P < 0.01, ***P < 0.001). c) Fluorescence images and H&E staining of tumor slices which were treated with CR760RAD‐NPs and CR760RGD‐NPs.

Based on the excellent optoacoustic properties on phantoms described above, CR760RGD‐NPs were next studied in 4T1 tumor models. Five 4T1 tumor‐bearing mice per treatment were intravenously (i.v.) injected with control CR760RAD‐NPs or CR760RGD‐NPs. **Figure** **3**a shows representative unmixed multispectral optoacoustic tomography (MSOT) images of 4T1 tumor‐bearing mice, acquired at different time points post‐injection (0, 1, 4, 8, and 24 h). The green signals represent the unmixed CR760 signal. As observed from the signal strength in the tumor regions, both CR760RAD‐NPs and CR760RGD‐NPs could effectively reach the tumor at the early time points (1 and 4 h), presumably due to the enhanced permeability and retention (EPR) effect. However, enabled by active tumor‐targeting, CR760RGD‐NPs remained in the tumor regions longer. At 24 h, the optoacoustic signal from the tumors treated with CR760RAD‐NPs was very weak compared to the tumors treated with CR760RGD‐NPs due to systemic clearance from the tumor vasculature (Figure 3b; and Figure S16, Supporting Information). To validate the results of the in vivo MSOT images, tumors were isolated from all animals 24 h after MSOT scanning. Fluorescence images of tumors sectioning from the two groups were acquired with a 750 nm excitation. Figure 3c shows a strong fluorescence signal from the CR760RGD‐NPs treated group but very weak fluorescence from the CR760RAD‐NPs treated group. Hematoxylin and eosin (H&E) staining confirmed the presence of tumors. To analyze the biodistribution of both types of nanoparticles, the vital organs (liver, kidney, heart, spleen) were also isolated 24 h after treatment and the optoacoustic measurements were performed using MSOT. The optoacoustic coronal plane images and corresponding optoacoustic signal intensities of different organs are shown in Figure S17 (Supporting Information). The two types of nanoparticles showed a similar level of uptake in the liver and a slightly lower uptake in the kidney, spleen, and heart.

### 2.5 In Vivo PTT Efficacy of CR760RGD‐NPs in a 4T1 Tumor Model

**Figure 4:**
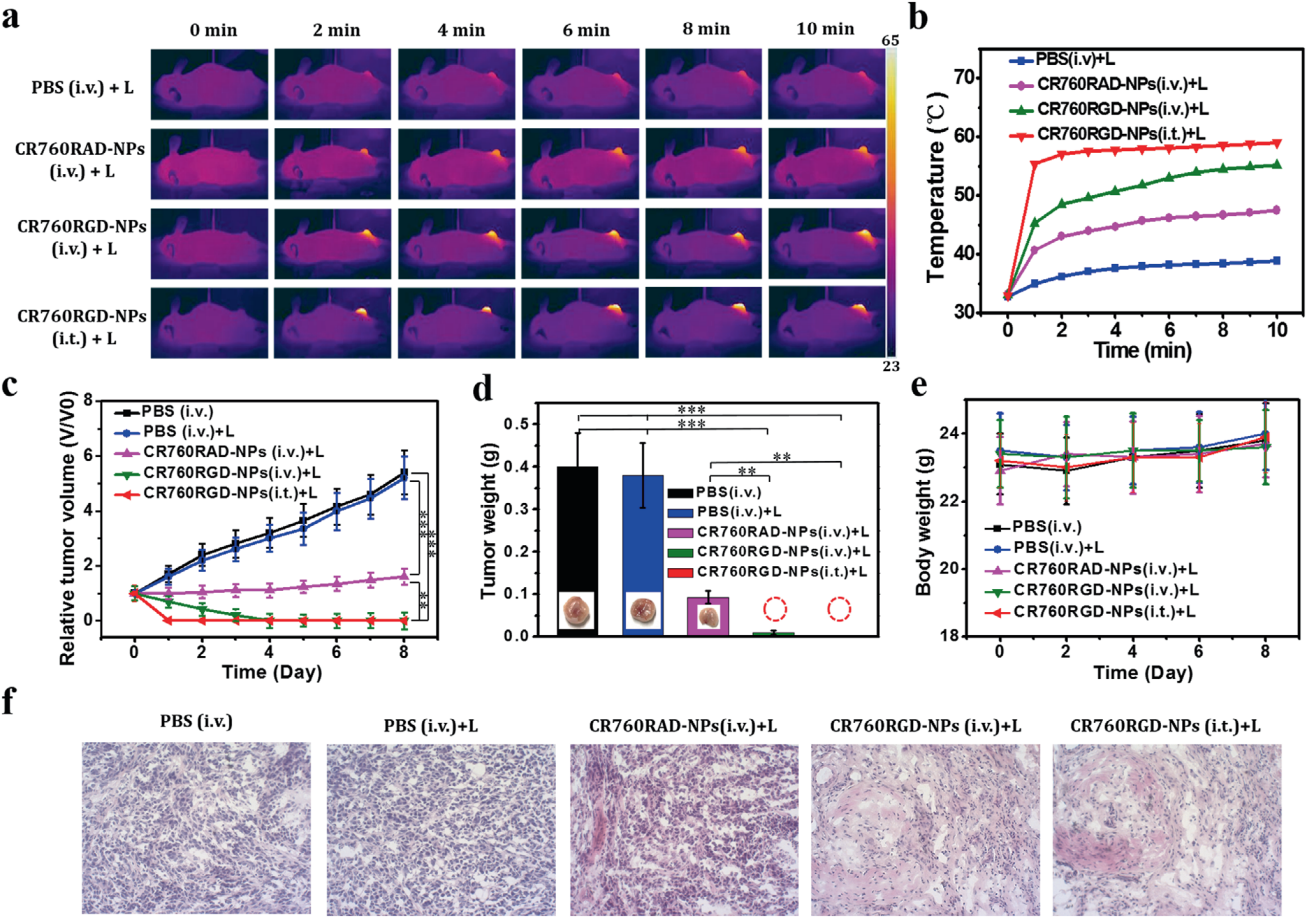
a) Infrared thermal images of 4T1 tumor‐bearing mice with different treatments exposed to a 780 nm laser (1 W cm^−2^) recorded at different time intervals. b) Temperature of tumors monitored by the infrared thermal camera in different groups upon laser irradiation as indicated in a). c–e) Relative tumor volumes, tumor weight, and body weight of mice treated with PBS, PBS (i.v.)+laser, CR760RAD‐NPs (i.v.)+laser, CR760RGD‐NPs(i.v.)+laser, and CR760RGD‐NPs(i.t.)+laser (*n* = 5, One‐Way ANOVA with Tukey's HSD test, **P < 0.01, ***P < 0.001). f) H&E staining of tumors isolated from mice at day 8 after various treatment. i.v. = intravenous, i.t. = intratumoral (20× magnification).

4T1 tumor‐bearing mice were randomized into five groups that received one of the following treatments: i) i.v. injection of 100 µL PBS, ii) i.v. injection of 100 µL PBS + laser irradiation, iii) i.v. injection of 100 µL CR760RAD‐NPs (1 × 10^−3^ m) + laser irradiation, iv) i.v. injection of 100 µL CR760RGD‐NPs (1 × 10^−3^ m) + laser irradiation v) intratumoral (i.t.) injection of 20 µL CR760RGD‐NPs (1 × 10^−3^ m) + laser irradiation. An irradiation time of 4 h postinjection was identified as being optimal for optoacoustic imaging. A thermal imaging camera recorded the temperature change of tumor regions from the treated groups until 10 min. As can be seen in **Figure** **4**a,b, the tumor temperature in the laser‐irradiated area treated intratumorally with CR760RGD‐NPs reached as high as 59 °C. Additionally, the temperature of the tumor region i.v. injected with CR760RGDs reached 55 °C, while the control groups treated with CR760RAD‐NPs or PBS reached 46.2 and 39.2 °C, respectively. We compared i.v. injected groups with i.t. injected group in which a known concentration of NPs was present in the tumor. This comparison was performed to mainly study the difference of PTT efficacy upon i.v. injection of targeted CR760RGD‐NPs against i.t. injected CR760RGD‐NPs. No significant difference in therapeutic efficacy between the i.v. injected and i.t. injected CR760RGD‐NPs was observed and indicated that targeted CR760RGD‐NPs at selected doses exhibit an effective PTT response independent of the administration mode.

During the subsequent 8‐day observation period, the tumor volumes and body weights of the four groups were measured daily. Group (v) with intratumoral injection of CR760RGD‐NPs displayed complete tumor elimination. Groups (iii) and (iv) showed significant inhibition of tumor growth, and due to the active tumor‐targeting of CR760RGD‐NPs, group (iv) exhibited almost complete tumor elimination. In contrast, the tumors from the other two control groups showed consistently high growth rates, suggesting that the PTT induced by CR760RGD‐NPs can significantly eliminate cancer cells (Figure 4c,d). No abnormalities in animal body weights were observed (Figure 4e). After 8 days, the tumors from each group were isolated and stained with H&E. The tumor slices from CR760RGD‐NPs (i.v., i.t.) + laser treated mice exhibited condensed nuclei and shallow staining color, which indicates cell apoptosis or necrosis (Figure 4f). Collectively, these results suggested that CR760RGD‐NPs can be an efficient PTT agent for cancer treatment.

### 2.6 In Vivo Pharmacokinetics and Biosafety of CR760RGD‐NPs

**Figure 5:**
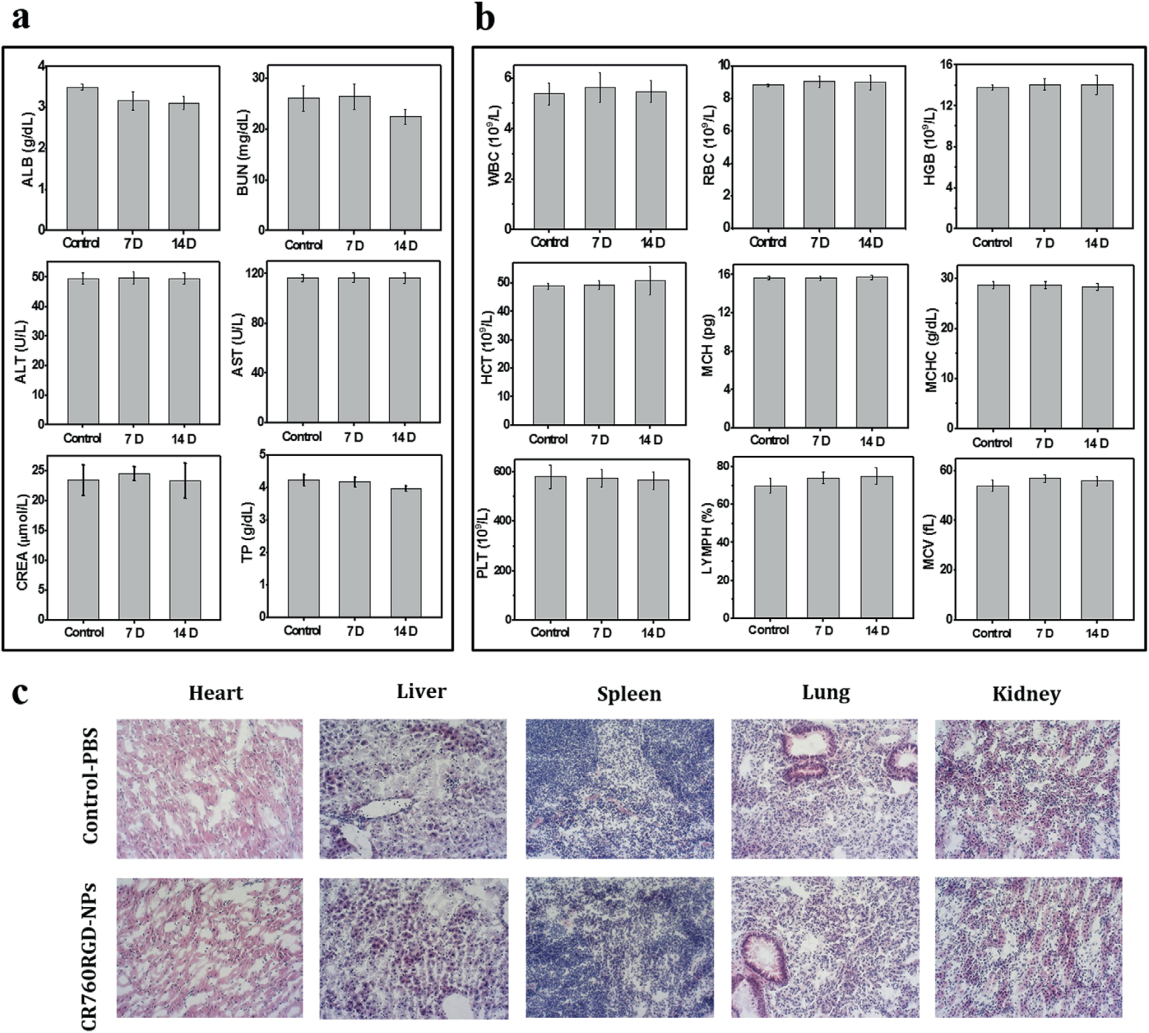
a,b) Blood biochemistry and hematology data of healthy C57BL/6 mice after i.v. injection of CR760RGD‐NPs (1 × 10^−3^ m) or PBS (control) (*n* = 5). ALB, albumin; BUN, blood urea nitrogen; ALT, alanine transferase; AST, aspartate transferase; CREA, creatinine; TP, total protein; WBC, white blood cells; RBC, red blood cells; HGB, hemoglobin; HCT, hematocrit; MCH, mean corpuscular hemoglobin; MCHC, mean corpuscular hemoglobin concentration; PLT, platelet; LYMPH, lymphocytes; MCV, mean corpuscular volume. c) H&E staining of vital organs of mice treated after 14 days (20× magnification).

We first evaluated the blood clearance profile by intravenously injecting CR760RGD‐NPs into healthy mice.^[^ ^18^ ^]^ Figure S18 (Supporting Information) shows the blood CR760RGD‐NPs concentration versus time curve, indicating that the serum half‐life of CR780RGD‐NPs is 8.22 h. Furthermore, healthy C57BL/6 mice were injected with 100 µL of CR760RGD‐NPs (1 × 10^−3^ m) and blood samples were collected on day 7 and 14 postinjection for organ function examination (**Figure** **5**a,b). The blood chemistry parameters and hematology analysis results showed no noticeable difference between CR760RGD‐NPs treated mice and the PBS‐treated control mice. The H&E staining and histology analysis of vital organs (Figure 5c) further confirmed no evident cytotoxicity or damage to the organs in the treated group. These results provide no evidence of acute toxicity and suggest that CR760RGD‐NPs may be well tolerated and suitable for in vivo imaging and phototherapy.

## 3 Discussion

Here we describe the generation of CR760‐derived nanoparticles and demonstrate them as highly efficient optoacoustic and PTT agents for theranostics studies. CR760 can be synthesized in a single step with high purity, and was monoconjugated to a PEG linker to enable self‐assembled nanoparticles. The CR760 dye and self‐assembled CR760RGD‐NPs exhibited high OGE, high PCE, and good photostability when compared to the FDA‐approved gold standard ICG. Based on the EPR effect and *α*_V_ *β*_3_ targeting, CR760RGD‐NPs could effectively accumulate in the tumor tissue and exhibited strong optoacoustic signals. Furthermore, the strong photothermal performance of CR760RGD‐NPs not only ensured high‐efficiency cell‐ablating capability, but also drove complete tumor elimination in mice, highlighting its phototherapeutic performance. Moreover, systemically administered CR760RGD‐NPs showed no signs of systemic or organ‐specific toxicity.

While many organic dyes possess limited solubility, hindering their biomedical applicability, these limitations can be addressed by nanoformulations. We have previously developed PEGylated liposomes incorporating ICG, which displayed efficient tumor accumulation as visualized by the MSOT system.^[^ ^19^ ^]^ Furthermore, we employed a flash nanoprecipitaion method to encapsulate the iBu‐TSBSH5 dye into QH2 nanoparticles.^[^ ^20^ ^]^ Intrinsically self‐assembled nanoparticles have recently attracted much attention because of their ability to achieve a robust nanostructure with high dye conjugation. An expedient self‐assembly nanoagent involves covalent linkage to PEG, an FDA‐approved hydrophilic polymer, such as PEGylated cypate‐derived nanoparticles,^[^ ^15^ ^]^ PEGylated IR780 nanoparticles,^[^ ^21^ ^]^ and PEGylated porphyrin nanoparticles.^[^ ^22^ ^]^ In the current study, based on the hydrophilic PEG linker and hydrophobic dye interactions, we obtained self‐assembled CR760RGD‐NPs by covalent conjugation of the CR760 dye with NH_2_‐PEG2000‐SH and c(RGDyC). The ultrasmall size distribution (around 23 nm) allows these nanoparticles to accumulate at the tumor site via the EPR effect. Further modification with the RGD peptide assures active cancer‐targeting and enhanced retention, suitable for longitudinal imaging.

Agents exhibiting a high absorption cross‐section can enhance the contrast in visualizing and quantifying diverse biological processes and diseases by optoacoustic imaging.^[^ ^4b^ ^]^ In general, an ideal optoacoustic contrast agent should have the photophysical characteristics of a narrow NIR absorption peak, high molar extinction coefficient, low quantum yield, high OGE, good photostability, tumor specificity, and biocompatibility.^[^ ^23^ ^]^ OGE values represent the ability of the compound to convert optically absorbed energy into pressure waves that give a strong optoacoustic signal. CR760RGD‐NPs exhibit a superior OGE compared to other commercially available dyes. The strong photostability of CR760RGD‐NPs ensures continuous and accurate signal detection. Based on these promising optoacoustic properties and active tumor‐targeting, CR760RGD‐NPs efficiently accumulate in the tumor region and can be detected by MSOT with high sensitivity.

The 45.37% PCE of CR760RGD‐NPs is greater than most reported PTT agents.^[^ ^7^, ^24^ ^]^ Moreover, CR760RGD‐NPs generate a very low fluorescence signal, which quenches the radiative decay of fluorescence and enhances the nonradiative energy in the form of heat. Additionally, CR760RGD‐NPs do not generate singlet oxygen to triplet state intersystem crossing, which indicates lack of a photodynamic effect under laser irradiation. Therefore, CR760RGD‐NPs can serve as efficient PTT agents for tumor targeting and elimination.

In summary, CR760‐derived nanoparticles were developed for efficient optoacoustic imaging and PTT of tumors. The self‐assembled CR760RGD‐NPs displayed efficient tumor targeting, while the high OGE and photostability ensured strong optoacoustic contrast. The promising photothermal performance enables phototherapeutic efficacy in vitro and in vivo, while the absence of cytotoxic effects in major organs indicates the possibility for clinical translation. Therefore, CR760RGD‐NPs provide a valuable approach to construct smart theranostic platforms for future clinical applications.

## 4 Experimental Section

### Materials

Croconic acid, 2,3,3‐trimethyl‐3H‐indole‐5‐carboxylic acid, n‐butanol, toluene, and 9,10‐dimethylnathracene were bought from abcr GmbH (Germany). c(RADyC) and c(RGDyC) peptides were purchased from GL biochem (Shanghai, China). NH_2_‐PEG2000‐SH were obtained from Creative PEGWorks (Chapel Hill, USA). Indocyanine green (ICG), 2’,7‐dichlorodihydrofluorescein diacetate (H_2_DCFDA), and 3‐(4,5‐dimethylthiazol‐2‐yl)‐2,5‐diphenyltetrazolium bromide (MTT) were purchased from Sigma‐Aldrich (Munich, Germany). IRDye800CW NHS Ester was bought from LI‐COR Biosciences (Nebraska, USA). Calcein‐acetoxymethyl (AM) and ethidium homodimer‐1 (EthD1) were bought from Thermo Fisher Scientific. Other chemical reagents were obtained from abcr GmbH (Germany).

### Synthesis of CR760

Croconic acid, 142.1 mg (1 mmol), and 2,3,3‐trimethyl‐3H‐indole‐5‐carboxylic acid 385.8 mg (2 mmol) were dissolved in 1:1 anhydrous toluene/n‐butanol (20 mL) and heated to reflux overnight. After cooling to room temperature, the product was collected by filtration, washed with isopropanol, sonicated in EtOAc and the filtrate collected to give the title product as a black solid (394 mg, 81%). ^1^H NMR (400 MHz, DMSO‐*d*_6_) *δ*: 8.12 (s, 2H), 7.99 (d, J = 8.1Hz, 2H), 7.65–7.55(m, 2H), 6.06(s, 2H), 1.54(s, 12H). MALDI‐TOF: m/z = 513.175 [M + H]^+^.

### Synthesis of CR760‐PEG‐RAD and CR760‐PEG‐RGD

CR760 (0.1 mmol), EDC·HCl (0.12 mmol), and NHS (0.12 mmol) were stirred in DMF (1 mL) at room temperature for 2 h, after which time NH_2_‐PEG2000‐SH (0.1 mmol) was added, and the mixture was stirred for a further 24 h. The crude mixture was directly purified by C18 reversed phase chromatography. Then c(RADyC) (0.1 mmol) was reacted with the above samples in PBS buffer (pH 6.0) for 2 h and purified on a reverse‐phase HPLC column. CR760‐PEG‐RGD was synthesized in a similar way.

### Preparation of CR760RAD‐NPs and CR760RGD‐NPs

CR760‐PEG‐RAD (1 mg) or CR760‐PEG‐RGD (1 mg) was dissolved in 1 mL deionized water and sonicated for 5 min, resulting in the nanometer‐sized CR760RAD‐NPs or CR760RGD‐NPs.

### Characterization

^1^H NMR spectrum was measured on a Bruker spectrometer at 400 MHz. The m/z ratios of the compounds were recorded with a MALDI UltrafleXtreme (Bruker) using dihydroxybenzoic acid as the matrix. The molar absorption coefficient and quantum yield of CR760 were calculated as previously described.^[^ ^25^ ^]^ CR760RAD‐NPs and CR760RGD‐NPs suspended in deionized water were analyzed for morphology by transmission electron microscopy (Zeiss Libra 120 Plus, Carl Zeiss NTSb GmbH, Oberkochen, Germany) and for size and zeta potential by Malvern Zetasizer. Absorption spectra were measured with a UV‐1800 spectrometer (Shimadzu, Japan). Optoacoustic spectra of samples were carried out using an MSOT inVision 256‐TF (iThera Medical, Munich, Germany) and done the correction with India ink and the normalization with Brilliant Black BN (BBN).^[^ ^26^ ^]^ Briefly, the Indian ink was used to correct the known pulse‐to‐pulse fluctuations from the OPO laser. The BBN was selected as reference because its photostability and lack of any fluorescence generation allowed to assign an OGE value of 1.0, assuming no decay channels taking place. The OGE of samples is equal to the slope of the line where corrected optoacoustic intensities are plotted against absorbance.^[^ ^26^ ^]^ The photostability of samples was detected by an MSOT inVision 256‐TF with pulsed laser irradiation for 30 min (fluence 10 mJ cm^−2^).

### Photothermal Effect of CR760RGD‐NPs

The photothermal conversion abilities of CR760RGD‐NPs were evaluated by recording their temperature changes at different concentrations of CR760RGD‐NPs upon exposure to a 780 nm CW laser at different laser power using an IR thermal camera. The PCE of CR760RGD‐NPs was calculated from the reference.^[^ ^27^ ^]^ Briefly, the aqueous solution of CR760RGD‐NPs with a 0.61 optical absorbance value at 780 nm were subjected to a 780 nm CW laser (Thorlabs, Inc.) at a power density of 0.75 W cm^−2^ for 10 min and then removed from laser irradiation and air‐cooled to room temperature. The temperature changes of CR760RGD‐NPs throughout the whole process were recorded using the IR thermal camera. After that, the PCE of CR760RGD‐NPs was calculated by applying all data acquired above into the equation *η* = [(*hS*(*T*‐*T*_surr_)‐*Q*_Dis_]/*I*(1–10^−A780^), where *h* is the heat‐transfer coefficient, *S* is the surface area of the container, *T* is the sample temperature, *T*_surr_ is room temperature, *Q*_Dis_ is the heat dissipation due to the light absorbed by the quartz sample cell, *I* is laser power, and A780 is the absorbance value of sample at 780 nm.

### In Vitro Cell Uptake

4T1 cells (2 × 10^5^) were cultured in 6‐well plate containing sterile cover slips, and then treated with CR760RAD‐NPs or CR760RGD‐NPs (20 × 10^−6^ m) for 4 h. Cover slips were mounted on glass slides with mounting medium‐DAPI (Invitrogen). The prepared slides were imaged using Leica SP8 confocal microscope (Wetzlar, Germany).

### In Vitro PTT and ROS Generation

4T1 cells (1 × 10^4^ per well) were subcultured in a 96‐well plate overnight and then incubated with PBS, CR760RAD‐NPs (20 × 10^−6^ m), and CR760RGD‐NPs (20 × 10^−6^ m) for 4 h, followed by laser irradiation (780 nm CW laser, 1 W cm^−2^) for 5 min. Afterward, the cells were cultured for another 24 h to calculate the relative cell viabilities using the standard MTT assay. Live/dead cell assays were identified with calcine‐AM and EthD1. Briefly, 4T1 cells were seeded in 96 well plates and divided based on the different treatments. After 24 h, the cells were costained with Calcein‐AM and EthD1 for 30 min. Finally, the cells were rinsed with PBS and imaged with Leica DMI3000 B Inverted Microscope (Wetzlar, Germany). In vitro ROS generation was detected using H_2_DCFDA, which emits green fluorescence after encountering ROS. Briefly, 4T1 cells were coincubated with PBS or CR760RGD‐NPs (20 × 10^−6^ m) and H_2_DCFDA (20 × 10^−6^ m) for 4h. Then the cells were washed with PBS and treated with a laser (780 nm CW laser, 1 W cm^−2^) for 5 min. The fluorescence images for each group were captured immediately using Leica DMI3000 B Inverted Microscope.

### Mice Tumor Model

The 4T1 tumor models were prepared using nude female mice (6 weeks, Envigo, Germany). 4T1 cells (1 × 10^6^) in PBS (30 µL) were implanted on the back of the animals. In vivo optoacoustic imaging and PTT studies were carried out when the tumor size reached ≈100 mm^3^. All procedures involving animal experiments were approved by the Government of Upper Bavaria (ROB 55.2‐2532.Vet_02‐17‐178).

### In Vivo Optoacoustic Imaging

4T1 tumor‐bearing mice (*n* = 5) per treatment were i.v. injected with control CR760RAD‐NPs or CR760RGD‐NPs (1 × 10^−3^ m). In vivo optoacoustic images were acquired at different time points before and after injection (0, 1, 4, 8, 24 h) using an MSOT inVision 256‐TF (iThera Medical, Munich, Germany). The MSOT scanning was operated from 680 to 900 nm with a step size of 10 nm and 10 signal averages. The light was illuminated on the sample uniformly from 5 different directions using a one to ten (five pairs) fiber bundle. The generated acoustic signals were acquired using a 256‐element transducer array (5 MHz center frequency) with 270° angle coverage and 40 Ms s^−1^ DAQ sampling rate. The acquired sinogram data were initially filtered using a Chebyshev filter having the bandwidth as 0.1‐–8 MHz. The filtered sinogram data were used to reconstructing the mice/phantom image using a model‐based reconstruction with a least squares QR inversion method running for 100 iterations. The model‐based reconstruction was performed at all the wavelengths, i.e., 680–900 nm with a step size of 10 nm. The reconstructed images were used to detect the CR760 signal using the CR760 reference spectral information, the CR760 signal was detected using a linear regression approach (unmixing method). The averaged optoacoustic signals of tumor regions were extracted using ViewMSOT 4.0 software (iThera Medical, Munich).

### In Vivo PTT

The tumor‐bearing mice were randomly divided into five groups (*n* = 5 per group) and subjected to different treatments as follows: i) i.v. injection of 100 µL PBS, ii) i.v. injection of 100 µL PBS + laser irradiation, iii) i.v. injection of 100 µL CR760RAD‐NPs (1 × 10^−3^ m) + laser irradiation, iv) i.v. injection of 100 µL CR760RGD‐NPs (1 × 10^−3^ m) + laser irradiation iii) i.t. injection of 20 µL CR760RGD‐NPs (1 × 10^−3^ m) + laser irradiation. The thermal images and temperature changes of tumor regions for each group were monitored and recorded using an infrared thermal camera. The tumor volume and body weight were measured every day for 8 days, then the mice were sacrificed and the tumors from each group were resected for H&E staining.

### In Vivo Pharmacokinetic Studies

Healthy C57BL/6 mice were intravenously injected with 100 µL of CR760RGD‐NPs (1 × 10^−3^ m) (each time point, *n* = 3 mice). At different time points (10 min, 1, 2, 4, 8, and 24 h), the blood was collected from the mouse heart, and then serum was separated by centrifugation using serum separator tubes (Sarstedt AG &Co.). Absorption intensities of nanoparticles in serum samples were measured by a UV‐1800 spectrometer (Shimadzu, Japan). Pharmacokinetic parameters were calculated with noncompartmental analysis using WinNonlin 4.1 software (Pharsight Corp., Palo Alto, CA).

### Blood Hematology and Biochemistry Analyses

C57BL/6 mice were randomly separated into 3 groups (5 mice each group). The control group was i.v. injected with 100 µL of PBS buffer and blood was collected on day 14. The other 2 groups were i.v. injected with 100 µL of CR760RGD‐NPs (1 × 10^−3^ m) and blood was collected on days 7 and 14. Then hematology and blood biochemistry tests were carried out to analyze the detailed parameters of blood samples using the Hitachi 917 Clinical Chemistry Analyzer (Roche, Germany). The vital organs were isolated for H&E staining.

### Statistical Analysis

Animals of the same gender, age, and genetic background were randomized for grouping. Sample sizes (n) were chosen based on guidance from the literature. Statistical analyses were performed using OriginPro 8 (Northampton, Massachusetts, USA). Inter‐group differences were assessed for significance using One‐Way/Two‐Way ANOVA with Tukey's HSD test. Results were expressed as mean ± SD, and differences were considered significant if *p* < 0.05.

## Conflict of Interest

V.N. is a shareholder in iThera Medical GmbH, Munich, Germany. The remaining authors declare no competing interests.

## Supporting information

Supporting Information

## Data Availability

Research data are not shared.
