# Supplementary material for: Facile Synthesis of a Croconaine‐Based Nanoformulation for Optoacoustic Imaging and Photothermal Therapy
Source: Adv Healthc Mater. 2021 Mar 18;10(9):2002115. doi: 10.1002/adhm.202002115 (PMC11469157; doi:10.1002/adhm.202002115)
Supplement: Supplementary file 1 — Supporting Information [file ADHM-10-2002115-s001.pdf]

© 2021 Wiley-VCH GmbH

ADVANCED HEALTHCARE MATERIALS

Supporting Information

for *Adv. Healthcare Mater.*, DOI: 10.1002/adhm.202002115

Facile synthesis of a croconaine-based nanoformulation for optoacoustic imaging and photothermal therapy

Nian Liu, Patrick O'Connor, Vipul Gujrati, Dimitris Gorpas, Sarah Glasl, Andreas Blutke, Axel Walch, Karin Kleigrewe, Michael Sattler, Oliver Plettenburg, Vasilis Ntziachristos**

Supporting Information

Facile synthesis of a croconaine-based nanoformulation for optoacoustic imaging and photothermal therapy

Nian Liu, Patrick O'Connor, Vipul Gujrati,* Dimitris Gorpas, Sarah Glasl, Andreas Blutke, Axel Walch, Karin Kleigrewe, Michael Sattler, Oliver Plettenburg, Vasilis Ntziachristos*

N. Liu, Dr. V. Gujrati, D. Gorpas, Prof. V. Ntziachristos
Chair of Biological Imaging, School of Medicine, Technical University of Munich, Munich 81675, Germany.
E-mail: vipul.gujrati@tum.de *, v.ntziachristos@tum.de *

N. Liu, Dr. V. Gujrati, D. Gorpas, S. Glasl, Prof. V. Ntziachristos
Institute of Biological and Medical Imaging, Helmholtz Zentrum München (GmbH), Neuherberg 85764, Germany.

Dr. P. O'Connor, Prof. O. Plettenburg
Institute of Medicinal Chemistry, Helmholtz Zentrum München (GmbH), Neuherberg 85764, Germany.

Dr. P. O'Connor, Prof. M. Sattler
Institute of Structural Biology, Helmholtz Zentrum München (GmbH), 85764 Neuherberg, Germany

Dr. A. Blutke, Prof. A. Walch
Research Unit Analytical Pathology, Helmholtz Zentrum München (GmbH), Neuherberg 85764, Germany.

Dr. K. Kleigrewe
Bavarian Center for Biomolecular Mass Spectrometry (BayBioMS), Technical University of Munich, Freising 85354, Germany

Prof. M. Sattler
Bavarian NMR Center and Center for Integrated Protein Science Munich at Department Chemie, Technical University of Munich, Garching 85747, Germany

Prof. O. Plettenburg
Center for Biomolecular Research, Institute of Organic Chemistry, Leibniz Universität Hannover, Schneiderberg 1b, D-30167 Hannover, Germany

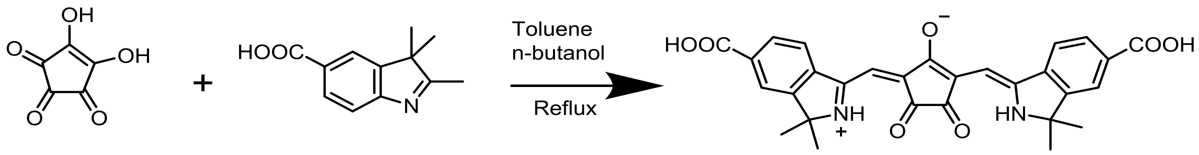

Figure S1 Schematic for synthesizing CR760.

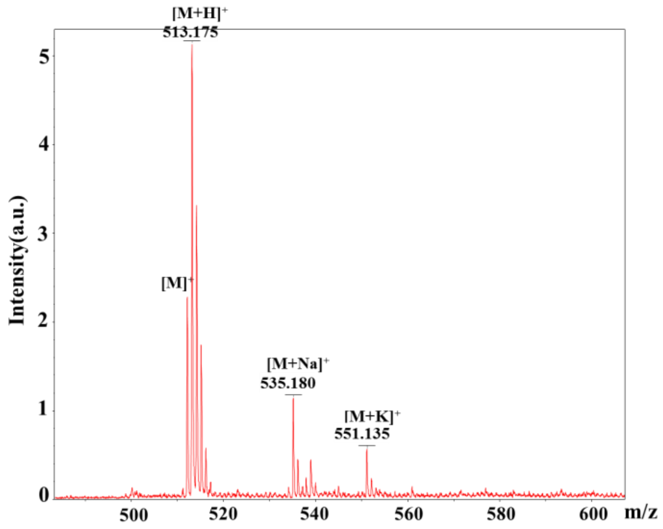

Figure S2 MALDI-TOF mass spectrum of CR760.

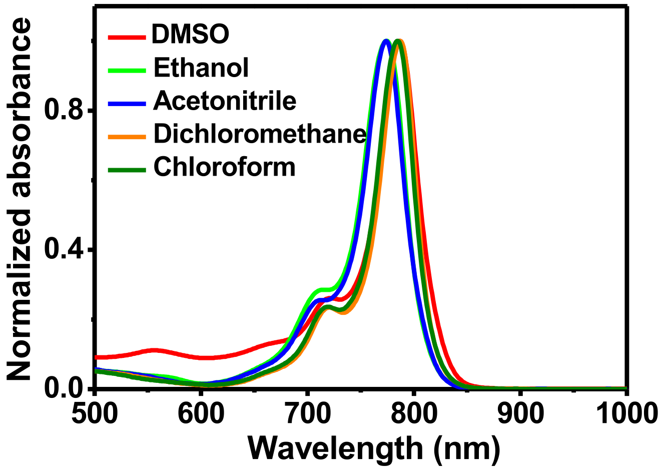

Figure S3 Normalized optical spectra of CR760 in different organic solvents.

Dye	Fluorescence Ex/Em (nm)	Molar absorption coefficient (M^{-1}	Quantum yield in ethanol	Optoacoustic generation

		cm^{-1}) in ethanol		efficiency in 10% FBS
**ICG**	780/810	2.1·10^5 at 780 nm	0.05	0.6855
**IRDye800CW**	780/800	2.54·10^5 at 780 nm	0.125	0.3741
**CR760**	760/790	3.25·10^5 at 780 nm	0.003	1.20

Table S1 Photo-physical properties of ICG, IRDye800CW, and CR760.

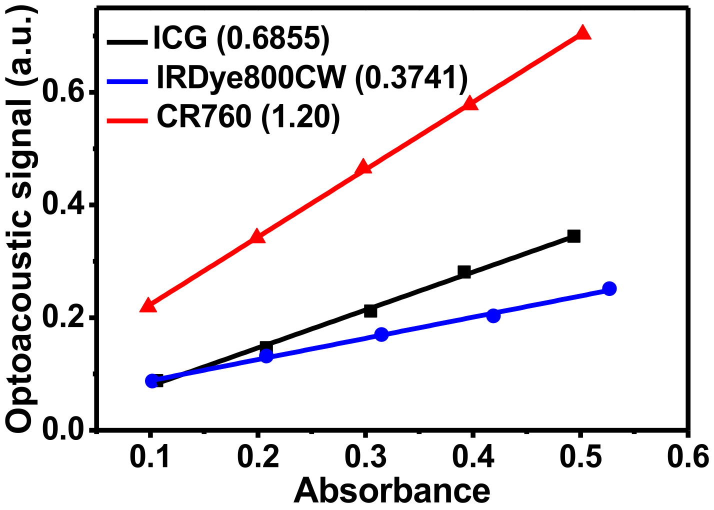

Figure S4 Optoacoustic signal intensities of ICG at 800nm, IRDye800CW at 780 nm, and CR760 at 760nm and different concentrations.

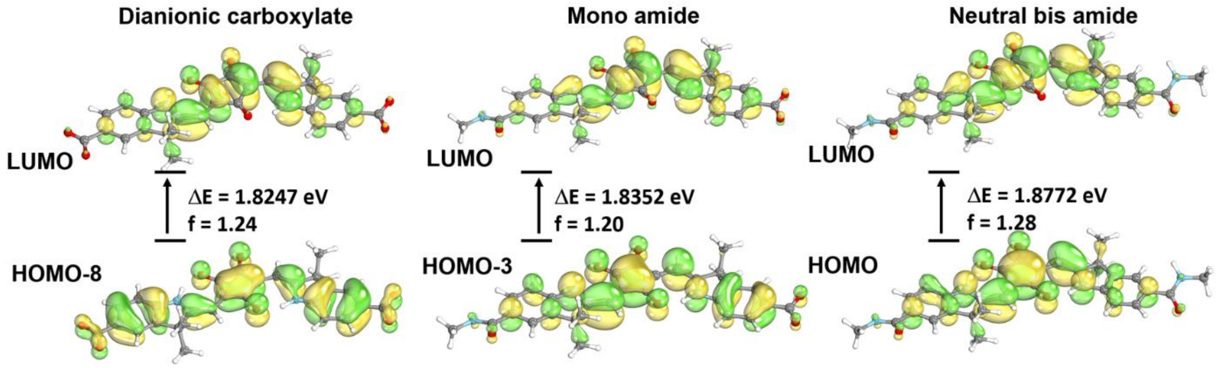

Figure S5 Molecular orbitals corresponding to major singlet π->π* excitations, energy levels, and corresponding oscillator strengths (f) for CR760 with neutral bis-amide, monoanion and dianion, gas phase calculations using Gaussian 16 Rev B.01.[1] Geometry optimizations and frequencies at the B3LYP/6-31G(d,p) level. UV absorption using TD-DFT at the B3LYP/cc-pVTZ level. Molecular orbitals appear to show a greater degree of charge transfer for ionized carboxylates relative to their neutral protonated congeners.

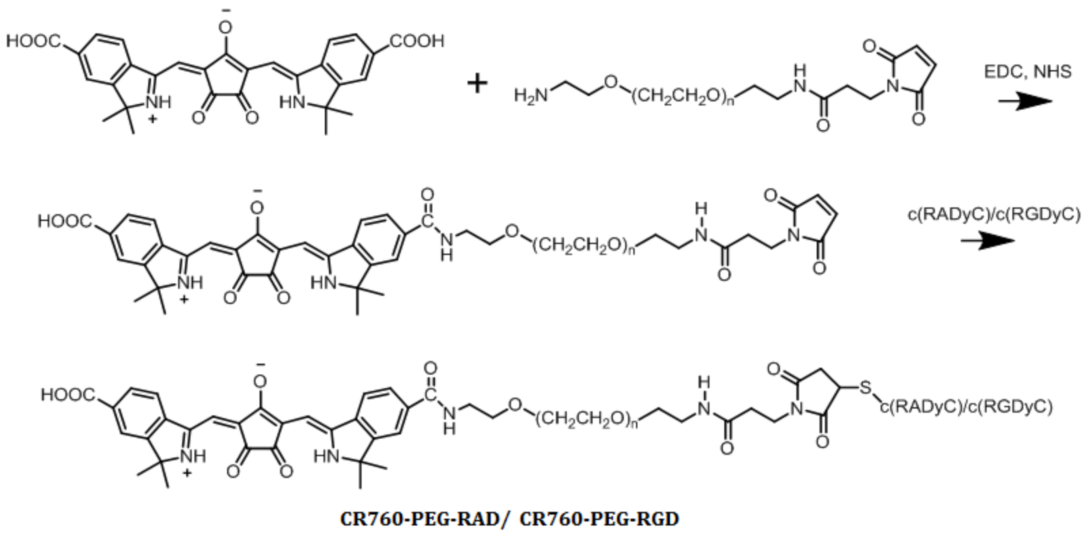

Figure S6 Schematic for synthesizing CR760-PEG-RAD/CR760-PEG-RGD.

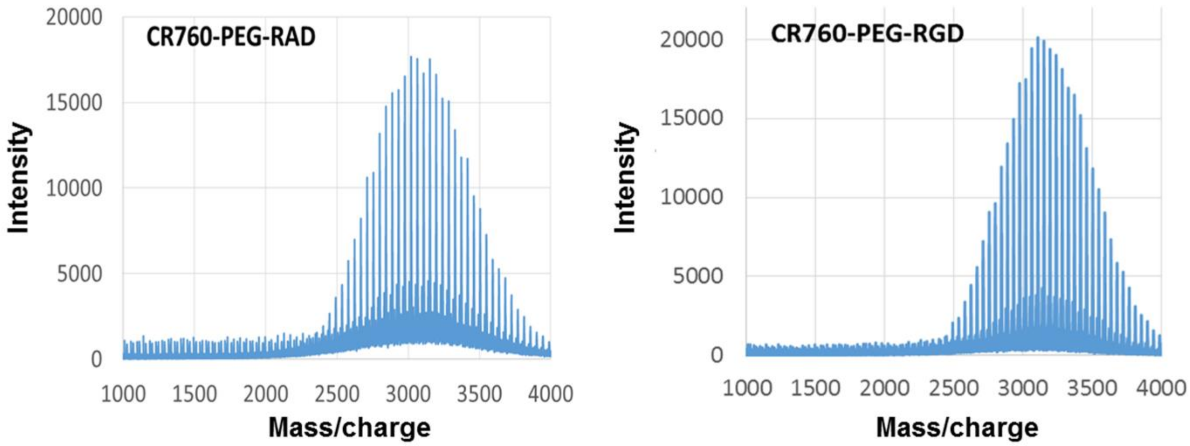

Figure S7 MALDI-TOF analyses of CR760-PEG-RAD and CR760-PEG-RGD.

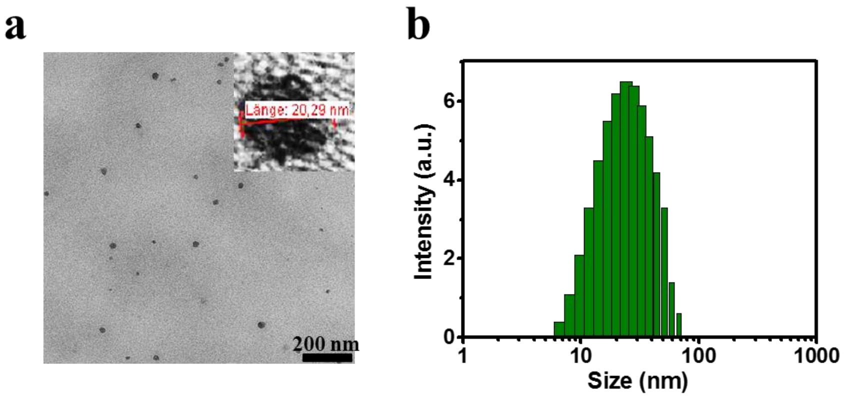

Figure S8 TEM image (a) and DLS profile (b) of CR760RAD-NPs (the insert represents the magnified TEM image).

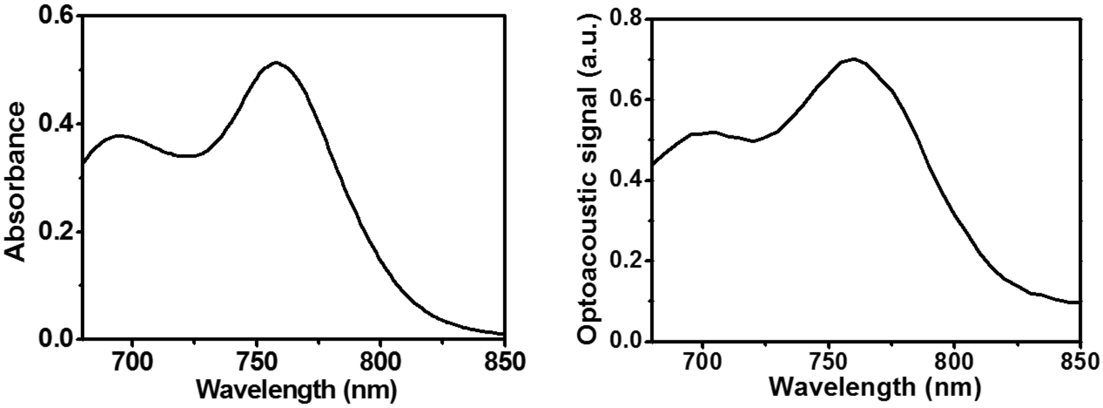

Figure S9 Optical spectrum and optoacoustic spectrum of CR760RAD-NPs.

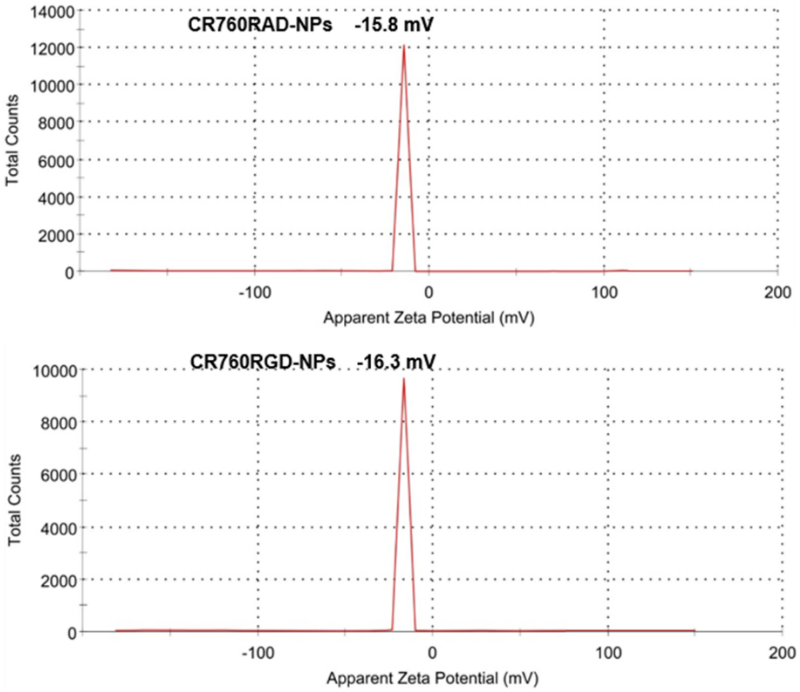

Figure S10 Zeta potential of CR760RAD-NPs and CR760RGD-NPs.

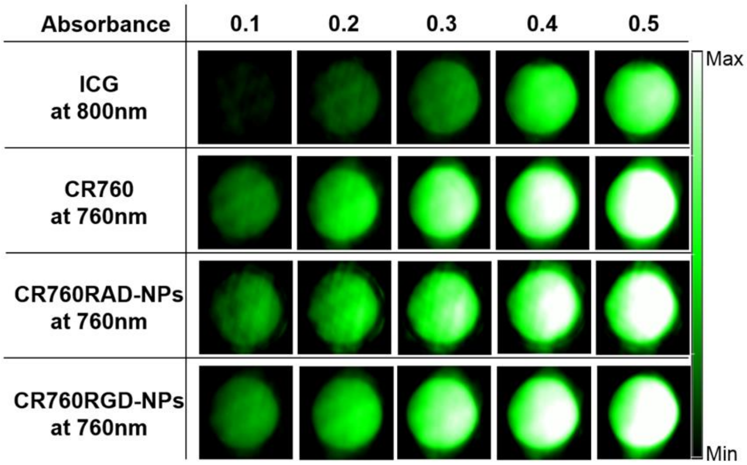

Figure S11 Optoacoustic phantom images of ICG, CR760, CR760RAD-NPs, and CR760RGD-NPs with different absorbance at their specific absorption peaks (ICG, 800nm; CR760, CR760RAD-NPs, CR760RGD-NPs, 760nm).

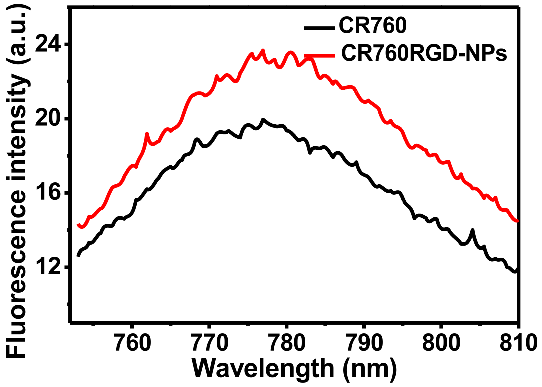

Figure S12 Fluorescence spectra of CR760 and CR760RGD-NPs (5μM) under 740nm excitation.

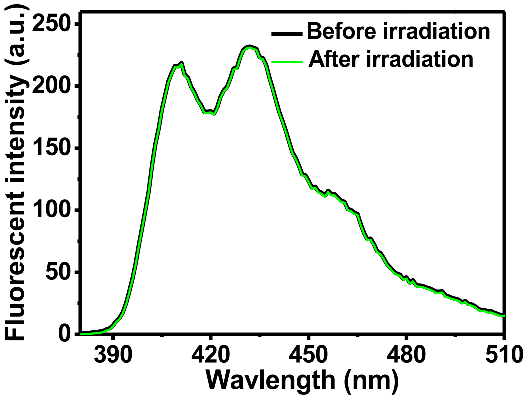

Figure S13 Fluorescent spectra of 9,10-dimethylnathracene (DMA) incubated with CR760RGD-NPs before and after laser irradiation. The fluorescent intensity shows no decrease after the treatment, which signifies no $^1O_2$ generation.

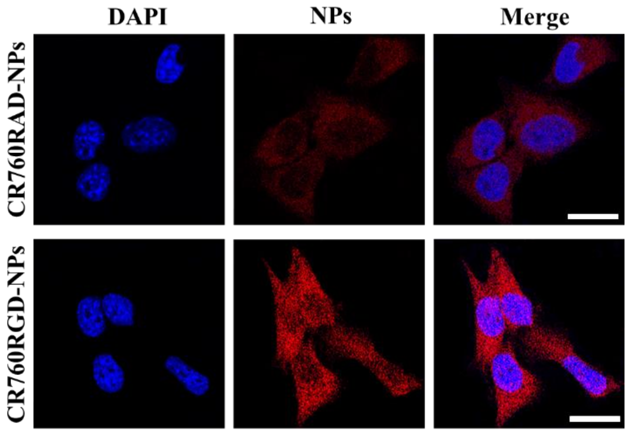

Figure S14 Cell uptake assay. Confocal microscope images of 4T1 cells treated with CR760RAD-NPs or CR760RGD-NPs for 4h. Scale bar, 25 μm.

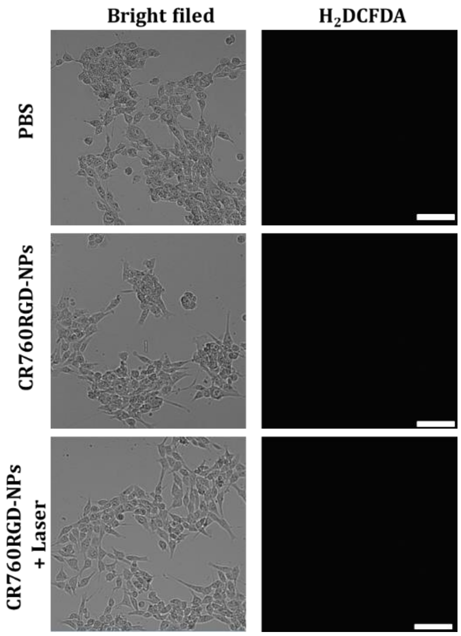

Figure S15 Fluorescence images of ROS generation in 4T1 cells with different treatment. Scale bar, 100 μm.

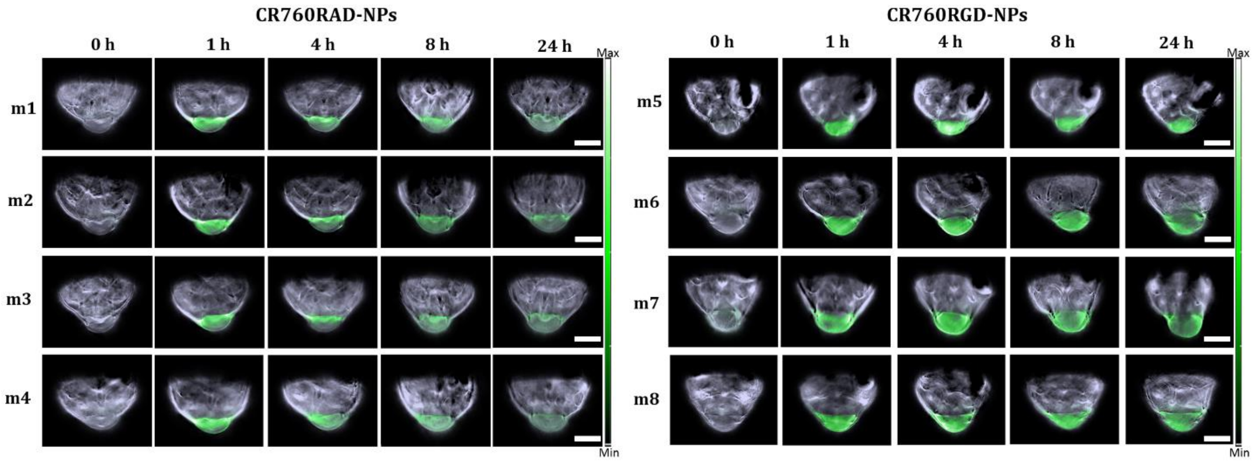

Figure S16 Representative unmixed MSOT images of 4T1 subcutaneous tumor models i.v. injected with 100 μL of CR760RAD-NPs or CR760RGD-NPs (1 mM) (another 4 mice for each group), scale bar = 5 mm. Unmixed CR760 signal is shown in the green color in the tumor region.

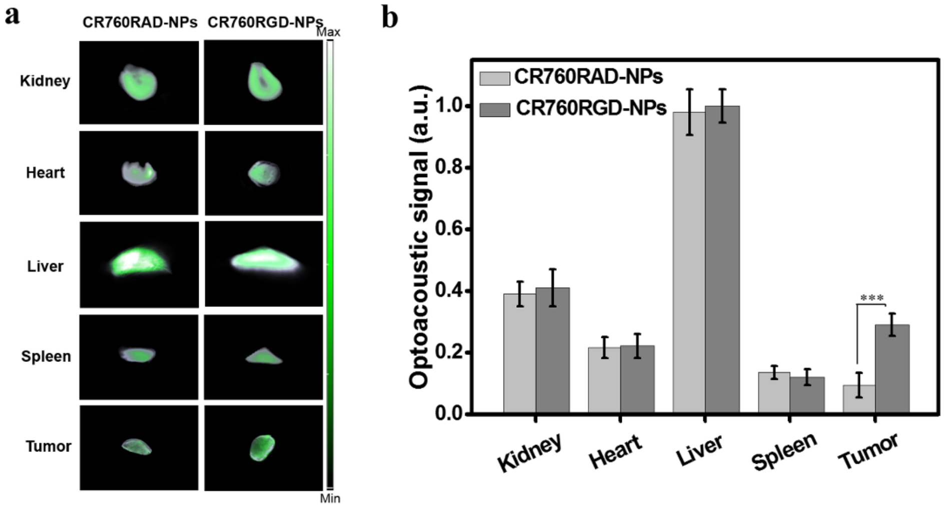

Figure S17 The biodistribution of CR760RAD-NPs and CR760RGD-NPs in vital organs at 24 h after intravenous injection into 4T1-bearing mice. Animals were injected with 100 μL of CR760RAD-NPs or CR760RGD NPs (1 mM). (a) Optoacoustic coronal plane images of major organs. (b) Optoacoustic signal intensity of major organs. (n=5, One-Way ANOVA with Tukey's HSD test, ***P < 0.001).

a

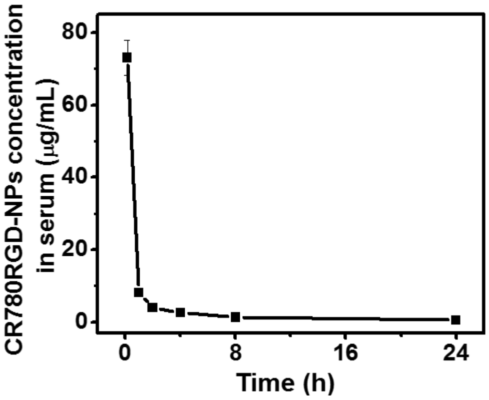

b

Parameter	
Cmax(μg/mL)	70.78
$T_{1/2z}$ (h)	8.22
AUClast(h*μg/mL)	70.81
Cl_obs (mL/h)	4.02

Figure S18 Pharmacokinetic analysis. (a) Blood CR760RGD-NPs concentration vs. time curve in healthy mice intravenously injected with 100 μL of CR780RGD-NPs (1 mM) (each time point, n=3 mice). **(**b**)** Pharmacokinetic parameters of CR760RGD-NPs intravenously administered to healthy C57BL/6 mice in (a). C_{max}, peak concentration; $T_{1/2z}$, elimination half-life; AUC, area under the curve; Clz, clearance.

[1] M. J. Frisch, G. W. Trucks, H. B. Schlegel, G. E. Scuseria, M. A. Robb, J. R. Cheeseman, G. Scalmani, V. Barone, G. A. Petersson, H. Nakatsuji, X. Li, M. Caricato, A. V. Marenich, J. Bloino, B. G. Janesko, R. Gomperts, B. Mennucci, H. P. Hratchian, J. V. Ortiz, A. F. Izmaylov, J. L. Sonnenberg, D. Williams-Young, F. Ding, F. Lipparini, F. Egidi, J. Goings, B. Peng, A. Petrone, T. Henderson, D. Ranasinghe, V. G. Zakrzewski, J. Gao, N. Rega, G. Zheng, W. Liang, M. Hada, M. Ehara, K. Toyota, R. Fukuda, J. Hasegawa, M. Ishida, T. Nakajima, Y. Honda, O. Kitao, H. Nakai, T. Vreven, K. Throssell, J. A. Montgomery, Jr, J. E. Peralta, F. Ogliaro, M. J. Bearpark, J. J. Heyd, E. N. Brothers, K. N. Kudin, V. N. Staroverov, T. A. Keith, R. Kobayashi, J. Normand, K. Raghavachari, A. P. Rendell, J. C. Burant, S. S. Iyengar, J. Tomasi, M. Cossi, J. M. Millam, M. Klene, C. Adamo, R. Cammi, J. W. Ochterski, R. L. Martin, K. Morokuma, O. Farkas, J. B. Foresman, D. J. Fox, Gaussian 16, Revision B.01 **2016** (Gaussian, Inc.: Wallingford, CT).
